# Supplementary material for: Difference in the Effect of Applying Bacillus to Control Tomato Verticillium Wilt in Black and Red Soil
Source: Microorganisms. 2024 Apr 15;12(4):797. doi: 10.3390/microorganisms12040797 (PMC11052436; doi:10.3390/microorganisms12040797)
Supplement: Supplementary file 1 [file microorganisms-12-00797-s001.zip › microorganisms-2945733-supplementary.pdf]

Difference in the Effect of Applying *Bacillus* to Control Tomato Verticillium Wilt in Black and Red Soil

Authors:

Zhenhua Guo[a, #], Ziyu Lu[a, #], Zhongwang Liu[a], Wei Zhou[a], Shuangyu Yang[a], Jiayan Lv[a], Mi Wei[a,]*

Affiliations:

[a] School of Agriculture, Shenzhen Campus, Sun Yat-Sen University, Shenzhen 518107, China.

[#] Zhenhua Guo and Ziyu Lu contributed equally to this article.

Contact information of co-author(s):

Zhenhua Guo, e-mail: 15546100532@163.com; Ziyu Lu, e-mail: luzy5@mail2.sysu.edu.cn; Zhongwang Liu, wangzhongliu668@163.com; Wei Zhou, zhouw79@mail2.sysu.edu.cn; Shuangyu Yang, ysy251147@163.com; Jiayan Lv, lvjy28@mail2.sysu.edu.cn; Mi Wei， e-mail: weim29@mail.sysu.edu.cn.

* Correspondence

ORCiD: 0000-0002-5761-3970

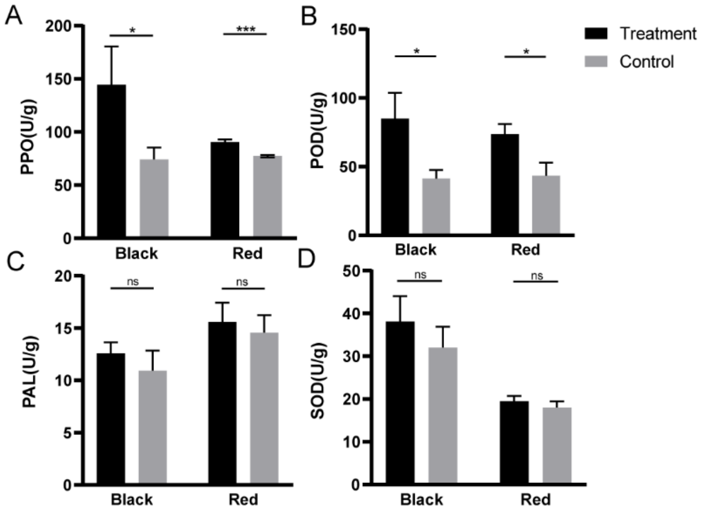

Figure S1. Effects of *Bacillus* on tomato resistance-related enzymes in black and red soils. (A) Polyphenol oxidase (PPO), (B) peroxidase (POD), (C) phenylalanine ammonia lyase (PAL), (D) superoxide dismutase (SOD).

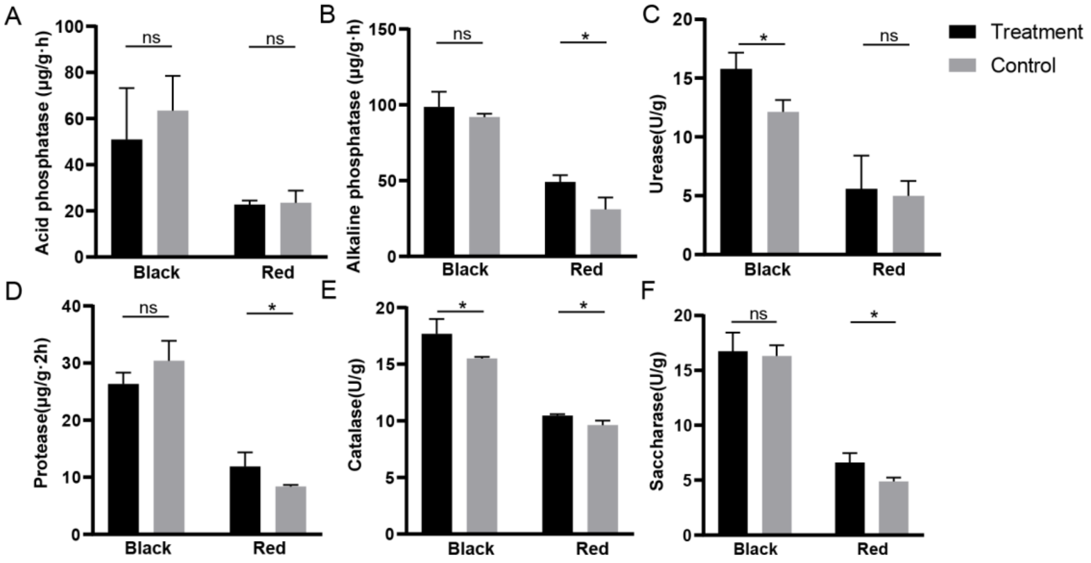

Figure S2. Effects of *Bacillus* on enzyme activities in soils with nutrient differences.

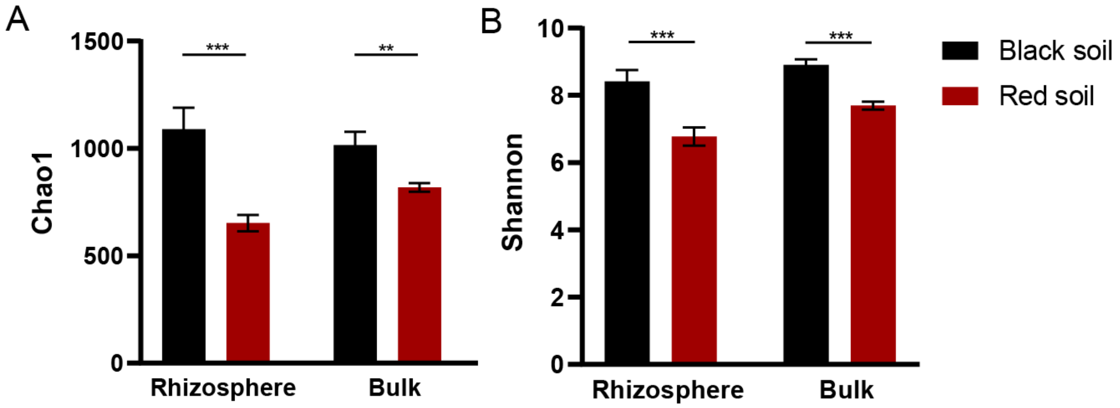

Figure S3. *Alpha* diversity of bacterial community in tomato roots in soil with nutrient differences.

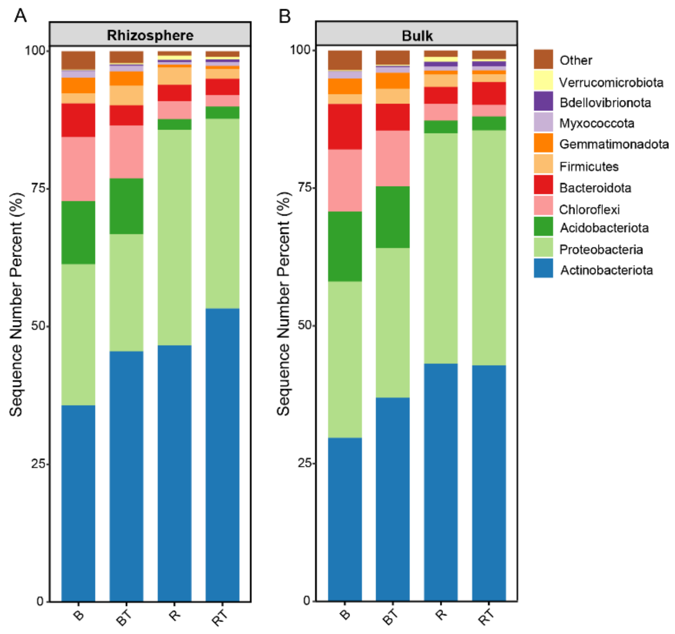

Figure S4. The percentage of bacteria microbial community composition at the phylum level in tomato rhizosphere and bulk soil under different treatments.

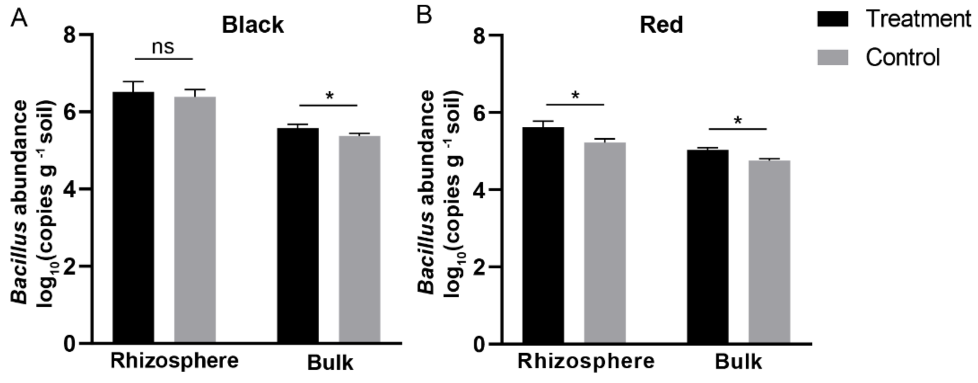

Figure S5. qPCR quantification of the genus *Bacillus* in the rhizosphere and non-rhizosphere of black and red soils under different treatments.

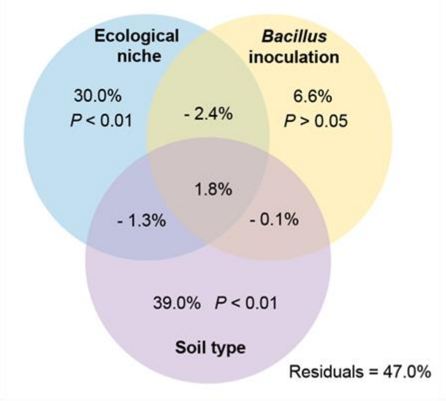

Figure S6. Effects of soil type, ecological niche, and exogenous *Bacillus* on root bacterial community of tomato.

Table S1. Basic physical and chemical properties of black and red soils.

	Black soil	Red soil
TN (mg/kg)	1392.33	212
TP (mg/kg)	745.33	364
TK (g/kg)	5.77	8.59
SOM (g/kg)	8.17	8.15
AP (mg/kg)	25.18	3.21
AK (mg/kg)	256.51	105.77
NH_4^+-N (mg/kg)	1.54	1.04
NO_3^--N (mg/kg)	87.2	2.07

Table S2. Evaluation of growth-promoting ability and biofilm formation ability of different microbial combinations.

Combination/ isolate	Inorganic phosphate	Organic phosphate	Nitrogen fixation	Potassium solublizatio n	IAA production	Biofilm formation
Combination A	+++	++	++	+++	+++	+++
Combination B	++	++	++	++	++	++
Combination C	+	++	+++	++	++	++
D2	++	-	+	+++	++	
ZJ-11	++	++	+++	-	++	
A#	+	+++	++	-	+++	
B#	+	+++	+++	-	+++	
2#	+	+++	+++	-	+++	
13-2	+++	-	-	+++	++	

Note: Combination A: D2+ZJ-11+13-2+A#; Combination B: B#+13-2+2#; Combination C: A#+B#+13-2+2#.

Table S3. Global network properties of bacteria molecular ecological networks among different treatments.

		BTR	BR	BTB	BB	RTR	RR	RTB	RB
Empirical	Total node	615	766	545	691	433	370	506	418

networks	Total links	3906	7336	3472	8356	2119	1163	1823	1802
	Avg degree (avgK)	12.702	19.154	12.741	24.185	9.788	6.286	7.206	8.622
	Connectedness (Con)	0.795	0.787	0.702	0.700	0.76	0.731	0.664	0.836
	Density	0.021	0.025	0.023	0.035	0.023	0.017	0.014	0.021
	Module	31	42	41	49	24	27	48	22
	Average clustering coefficient (avgCC)	0.376	0.354	0.325	0.315	0.353	0.197	0.193	0.277
	Average path distance (GD)	4.934	5.349	6.443	7.312	6.134	6.641	6.528	5.663
	Modularity	0.661	0.479	0.693	0.319	0.673	0.747	0.802	0.798
Random networks	Average clustering cofficient (avgCC)	0.093± 0.004	0.163± 0.005	0.085± 0.005	0.251± 0.007	0.082± 0.006	0.051± 0.006	0.043± 0.005	0.042± 0.004
	Average path distance (GD)	2.881± 0.015	2.733± 0.015	2.906± 0.018	2.685± 0.017	2.974± 0.019	3.336± 0.032	3.334± 0.026	3.071± 0.019
	Modularity	0.213± 0.003	0.154± 0.003	0.212± 0.004	0.115± 0.002	0.255± 0.004	0.351± 0.005	0.321± 0.004	0.293± 0.004
P	Average clustering cofficient (avgCC)	<0.0001		<0.0001		<0.0001		<0.0001	
	Average path distance (GD)	<0.0001		<0.0001		<0.0001		<0.0001	
